# Switching TNFα inhibitors: Patterns and determinants

**DOI:** 10.1002/prp2.843

**Published:** 2021-07-24

**Authors:** Rosanne W. Meijboom, Helga Gardarsdottir, Matthijs L. Becker, Mark C. H. de Groot, Kris L. L. Movig, Johan Kuijvenhoven, Toine C. G. Egberts, Hubert G. M. Leufkens, Thijs J. Giezen

**Affiliations:** ^1^ Pharmacy Foundation of Haarlem Hospitals Haarlem The Netherlands; ^2^ Division of Pharmacoepidemiology & Clinical Pharmacology Utrecht Institute for Pharmaceutical Sciences Utrecht The Netherlands; ^3^ Department of Clinical Pharmacy University Medical Centre Utrecht Utrecht The Netherlands; ^4^ Department of Pharmaceutical Sciences University of Iceland Reykjavik Iceland; ^5^ Department of Clinical Pharmacy Spaarne Gasthuis, Haarlem and Hoofddorp Haarlem The Netherlands; ^6^ Central Diagnostic Laboratory Division Laboratories, Pharmacy and Biomedical Genetics University Medical Centre Utrecht Utrecht The Netherlands; ^7^ Department of Clinical Pharmacy Medisch Spectrum Twente Enschede The Netherlands; ^8^ Department of Gastroenterology and Hepatology Spaarne Gasthuis, Haarlem and Hoofddorp The Netherlands

**Keywords:** biological products, drug utilization, inflammatory bowel diseases, pharmacoepidemiology, rheumatology, tumor necrosis factor inhibitors

## Abstract

The aim of this study was to assess switching patterns and determinants for switching in patients initiating TNFα inhibitor (TNFα‐i) treatment. Patients were included who started TNFα‐i treatment between July 1, 2012 and December 31, 2017, from three Dutch hospitals, and were diagnosed with rheumatic diseases (RD), inflammatory bowel disease (IBD), or psoriasis. Outcomes were switching, defined as initiating another biological; switching patterns including multiple switches until the end of follow‐up; determinants for first switch, assessed using multivariate logistic regression. A total of 2228 patients were included (median age 43.3 years, 57% female), of which 52% (*n* = 1155) received TNFα‐i for RD, 43% (*n* = 967) for IBD, and 5% (*n* = 106) for psoriasis. About 16.6% of RD patients, 14.5% of IBD patients, and 16.0% of psoriasis patients switched at least once, mainly to another TNFα‐i. TNFα‐i dose escalation (OR 13.78, 95% CI 1.40–135.0) and high‐dose corticosteroids initiation (OR 3.62, 95% CI 1.10–12.15) were determinants for switching in RD patients. TNFα‐i dose escalation (OR 8.22, 95% CI 3.76–17.93), immunomodulator initiation/dose escalation (OR 2.13, 95% CI 1.04–4.34), high‐dose corticosteroids initiation (OR 6.91, 95% CI 2.81–17.01) and serum concentration measurement (OR 5.44, 95% CI 2.74–10.79) were determinants for switching in IBD patients. Switching biological treatment occurred in about one in six patients. RD patients with TNFα‐i dose escalation and/or high‐dose corticosteroids initiation were more likely to switch. IBD patients with TNFα‐i or immunomodulator initiation/dose escalation, high‐dose corticosteroids initiation or serum concentration measurement were more likely to switch. These findings might help clinicians anticipating switching in TNFα‐i treatment.

**Abbreviations**
IBD: inflammatory bowel disease
IMID: immune‐mediated inflammatory diseases
JAK: Janus Kinase
MST: Medisch Spectrum Twente
RA: rheumatoid arthritis
RD: rheumatic diseases
TNF: tumor necrosis factor
TNFα‐I: TNFα inhibitor
UPOD: Utrecht Patient Oriented Database

## 1 INTRODUCTION

Tumor necrosis factor (TNF) α inhibitors have revolutionized the treatment of several immune‐mediated inflammatory diseases (IMID), such as rheumatic diseases (RD), inflammatory bowel disease (IBD), and psoriasis. Five TNFα inhibitors are currently available for patient care in Europe: adalimumab and infliximab are, among others, approved for RD, IBD, and psoriasis, etanercept and certolizumab pegol are approved for RD and psoriasis and golimumab is approved for RD and IBD.^1^, ^2^, ^3^, ^4^, ^5^

TNFα inhibitors are advised as first‐line biological treatment in IMID when conventional immunomodulator treatment, such as methotrexate or azathioprine, does not achieve sufficient clinical benefit. TNFα inhibitors may improve clinical signs and symptoms and make low disease activity and remission realistic objectives for patients suffering from IMIDs.^6^, ^7^, ^8^, ^9^, ^10^ However, although many patients benefit from TNFα inhibitor treatment, several patients experience a lack of efficacy or bothersome side effects.^11^, ^12^ For those patients, switching to another biological drug, or to a Janus Kinase (JAK) inhibitor, is recommended. The choice for switching to a second TNFα inhibitor or to a biological drug belonging to another mechanistic class depends on the indication of use and on the reason for switching. For example, the IBD guideline advices on switching based on response to the TNFα inhibitor, drug concentrations and presence of antibodies,^6^ whereas the rheumatoid arthritis (RA) guideline does not provide a strategy in choosing between another TNFα inhibitor or to a biological drug belonging to another mechanistic class.^7^

In clinical practice, switching to another biological treatment frequently occurs. A previous study in RD patients showed that 67% of the patients remained persistent users (percentage of patients on the same biological drug after 12 months of initiation)^13^ of their first TNFα inhibitor, 13% had switched to another biological drug (other TNFα inhibitor or biological belonging to another mechanistic class) and 20% had discontinued biological treatment.^14^ A study in IBD patients reported a 1‐year persistence of TNFα inhibitors of 48.5% for CD and 44.8% for UC. Switching to another biological drug occurred in 19.4% of CD and 20.3% of UC patients.^15^ One‐year persistence was higher in psoriasis patients; 77.4% of patients were persistent users, 17.5% had switched to another biological drug and 5.1% had discontinued biological treatment.^16^

Several determinants for TNFα inhibitor treatment discontinuation in IMID have been identified. For example, women are at a 1.3 to 1.8 times higher risk for discontinuation than men.^17^, ^18^, ^19^ Concomitant use of methotrexate decreases the risk of discontinuation in RD patients by 22%,^20^ and in psoriasis patients by 66.2%.^19^ The risk of TNFα inhibitor treatment discontinuation additionally increases by 1.4–6.0% per year with increasing age.^19^, ^20^

The aforementioned studies mainly focused on biological treatment discontinuation and determinants thereof, or only on the first biological treatment switch, in a single indication. However, little has been studied on the patterns of multiple switches of biological treatment across multiple indications and on determinants specifically for switching biological treatment. Data on switching patterns, including information on the type of biological drug, and more knowledge on determinants for switching may support more efficient treatment with biological drugs.

The aim of this study was to assess switching patterns and determinants associated with switching in patients who initiated TNFα inhibitor treatment for IMID between 2012 and 2017.

## 2 MATERIALS AND METHODS

### 2.1 Design and setting

This cohort study included patients from three large hospitals in the Netherlands: the Spaarne Gasthuis, the Medisch Spectrum Twente (MST), and the University Medical Center Utrecht (UMC Utrecht). The Spaarne Gasthuis and the MST are both large teaching hospitals; the UMC Utrecht is an academic teaching hospital.

Dispensing data from the outpatient pharmacy from the Spaarne Gasthuis, the MST and the UMC Utrecht were obtained from CompuGroup Medical (CGM). Hospital and laboratory data from the Spaarne Gasthuis and the MST were obtained directly from the hospital and pharmacy information systems, that is, through Epic (Spaarne Gasthuis) and Vipharma, and GLIMS (MST).

Hospital and laboratory data from the UMC Utrecht were obtained from the Utrecht Patient Oriented Database (UPOD). UPOD is an infrastructure of relational databases comprising data on patient characteristics, hospital discharge diagnoses, medical procedures, medication orders, and laboratory tests for all patients treated at the UMC Utrecht since 2004. UPOD data acquisition and management was in accordance with current regulations concerning privacy and ethics. The structure and content of UPOD are described in more detail elsewhere.^21^

Since January 1, 2012, all outpatient‐administered biological drugs have been exclusively dispensed by the outpatient pharmacy of the hospital where a patient is treated due to reimbursement regulations in the Netherlands. Consequently, the outpatient pharmacy contains a complete overview of all biological drugs used in the home setting.^22^

### 2.2 Study population

All new users of TNFα inhibitors (etanercept, infliximab, adalimumab, certolizumab, and golimumab), treated for RD, IBD, or psoriasis, between July 1, 2012, and December 31, 2017 (Spaarne Gasthuis and MST) or between January 1, 2013 and December 31, 2017 (UMC Utrecht) were included in the cohort. New users were defined as patients who had no use of any biological drug for RD, IBD, or psoriasis for at least 6 months prior to the date of inclusion. The date of the start of the first TNFα inhibitor within the study period was assigned as the patient's index date.

For all patients included, date of birth, gender, treatment indication defined as RD, IBD, or psoriasis (derived from the specialism of the prescriber of the TNFα inhibitor), type of biological drug, dose and dosing regimen, dispensing date (outpatient biological drugs), or administration date (biological drugs administered at the hospital ward), having TNFα inhibitor serum concentration or anti‐drug antibodies measured, use of immunomodulators and high‐dose corticosteroids were collected.

### 2.3 Switching patterns

For each patient treatment episodes were constructed, defined as the duration of use of a single type of biological drug over time. For outpatient biological drugs, this was the time between the first dispensing of that biological drug until the end of the duration of the last dispensing. For biological drugs administered at the hospital ward, this was the time between the first administration of that biological drug until the last administration plus the standard dosing interval. A maximum permissible gap of 90 days (outpatient biological drugs) or twice the length of the standard dosing interval (biological drugs administered at the hospital ward) was allowed to correct for potential temporary treatment interruptions (e.g., due to surgery or infections).

From these treatment episodes, switching patients were identified, defined as starting a treatment episode of another biological drug (or a JAK inhibitor) within the maximum permissible gap of the previous one. In addition, patients who did not switch were identified as persistent users (one treatment episode for the index TNFα inhibitor from the index date until the end of follow‐up or censoring) or discontinuers of biological treatment (no dispensing of the index TNFα inhibitor, without switching).

For the analysis of switching patterns, all biological (and JAK inhibitor) treatment switches were studied, including multiple treatment switches. Sankey diagrams were constructed to present switching patterns, stratified by indication (RD, IBD, or psoriasis). The number of patients who switched and median time until the switch were added to the diagram.

The following biological drugs were included in the analysis: abatacept, anakinra, belimumab, brodalumab, canakinumab, guselkumab, ixekizumab, rituximab, sarilumab, secukinumab, ustekinumab, vedolizumab (biological drugs), and baricitinib and tofacitinib (JAK inhibitors).

### 2.4 Determinants for switching

Determinants for switching from the first TNFα inhibitor to another biological drug (or JAK inhibitor) were explored in a nested case–control analysis. Cases were defined as patients who switched at least once during follow‐up. Patients who did not switch were included as controls. Up to four controls were randomly selected for each case by using incidence density sampling. Cases and controls were matched by the type of TNFα inhibitor at the index date, treatment in the same hospital and the date of initiation of treatment (± 3 months). Controls could be selected more than once, and patients who became cases could be selected as controls at earlier time points.

The following determinants for switching were explored: age at index date (continuous, years); gender (categorical); dose escalation of TNFα inhibitor within 60 days before the switch (yes or no); initiation or dose escalation of treatment with immunomodulator within 60 days before the switch (yes or no); initiation of treatment with high‐dose corticosteroids within 60 days before the switch (yes or no); and TNFα inhibitor serum concentration measurement (or anti‐drug antibodies for the specific TNFα inhibitor) within 60 days before the switch (yes or no).

Dose escalation of outpatient‐administered TNFα inhibitors was defined as having any increase in dose or shortening of dosing interval of the index TNFα inhibitor in the 60‐day period before the switch. For infliximab, dose escalations were defined as either a minimum 25% increase^23^ in dose in the 60‐day period before the switch or an increase in dosing interval of a minimum of 8 days to overcome rounding up and dose increases due to an increased weight of the patient or logistic issues.

The following immunomodulators were included: sulfazalazine, mesalazine, mercaptopurine, tioguanine, mycophenolic acid, leflunomide, ciclosporin, azathioprine, methotrexate, and hydroxychloroquine.

### 2.5 Data analysis

Descriptive statistics were used to present the baseline characteristics of the patients. Treatment patterns were presented in a Kaplan–Meier curve for persistent use of index TNFα inhibitor. Switch to another biological drug or JAK inhibitor and discontinuation of index TNFα inhibitor without switching were presented in cumulative incidence curves.

Determinants for switching were analyzed with conditional logistic regression, stratified per indication. All possible determinants were first analyzed univariately, and determinants with a *p*‐value of <.1 in the univariate analysis were analyzed using multivariate conditional logistic regression.

In a sensitivity analysis, the impact of changing the definition of new users was assessed by only including patients who did not use any biological drug for RD, IBD, or psoriasis 12 months prior to the date of inclusion. This was done to discriminate prevalent users of TNFα inhibitor from new users.

Data were analyzed using R version 3.6.1 (R Foundation for Statistical Computing).

## 3 RESULTS

A total of 2228 patients were included, with a median age of 43.3 years, 56.6% of the patients being female (Table 1). Of the included patients, 1155 (51.8%) were diagnosed with RD, 967 (43.4%) with IBD, and 106 (4.8%) with psoriasis. Adalimumab was the most frequently (40.9%) used TNFα inhibitor for the total study population, but etanercept was the most used TNFα inhibitor in RD patients (47.5%), infliximab in IBD patients (62.4%). At baseline, 49.6% of the patients additionally used an immunomodulator. This differed between indications; with concomitant use in 58.1% of RD patients, 43.1% of IBD patients, and 16.0% of psoriasis patients.

**TABLE 1:** Patient characteristics of the cohort at baseline

	Total	RD	IBD	Psoriasis
No patients (%)	2228	1155	967	106
Females (%)	1261 (56.6%)	705 (61.0%)	515 (53.3%)	41 (38.7)
Median age (IQR) (years)	43.3 (26.8–57.2)	49.1 (33.3–70.0)	34.4 (22.5–51.2)	50.6 (34.4–60.4)
Etanercept	573 (25.7%)	549 (47.5%)	—	24 (22.7%)
Infliximab	654 (29.3%)	41 (3.6%)	603 (62.4%)	10 (9.4%)
Adalimumab	911 (40.9%)	488 (42.3%)	351 (36.3%)	72 (67.9%)
Certolizumab	13 (0.6%)	13 (1.1%)	—	—
Golimumab	77 (3.5%)	64 (5.5%)	13 (1.3%)	—
Baseline use of immunomodulator	1105 (49.6%)	671 (58.1%)	417 (43.1%)	17 (16.0%)
Median follow‐up (IQR) (years)	3.4 (2.1–5.0)	3.6 (2.2–5.2)	3.3 (2.0–4.8)	3.2 (2.1–4.5)

Abbreviation: IQR, interquartile range.

### 3.1 Switching patterns

**FIGURE 1:**
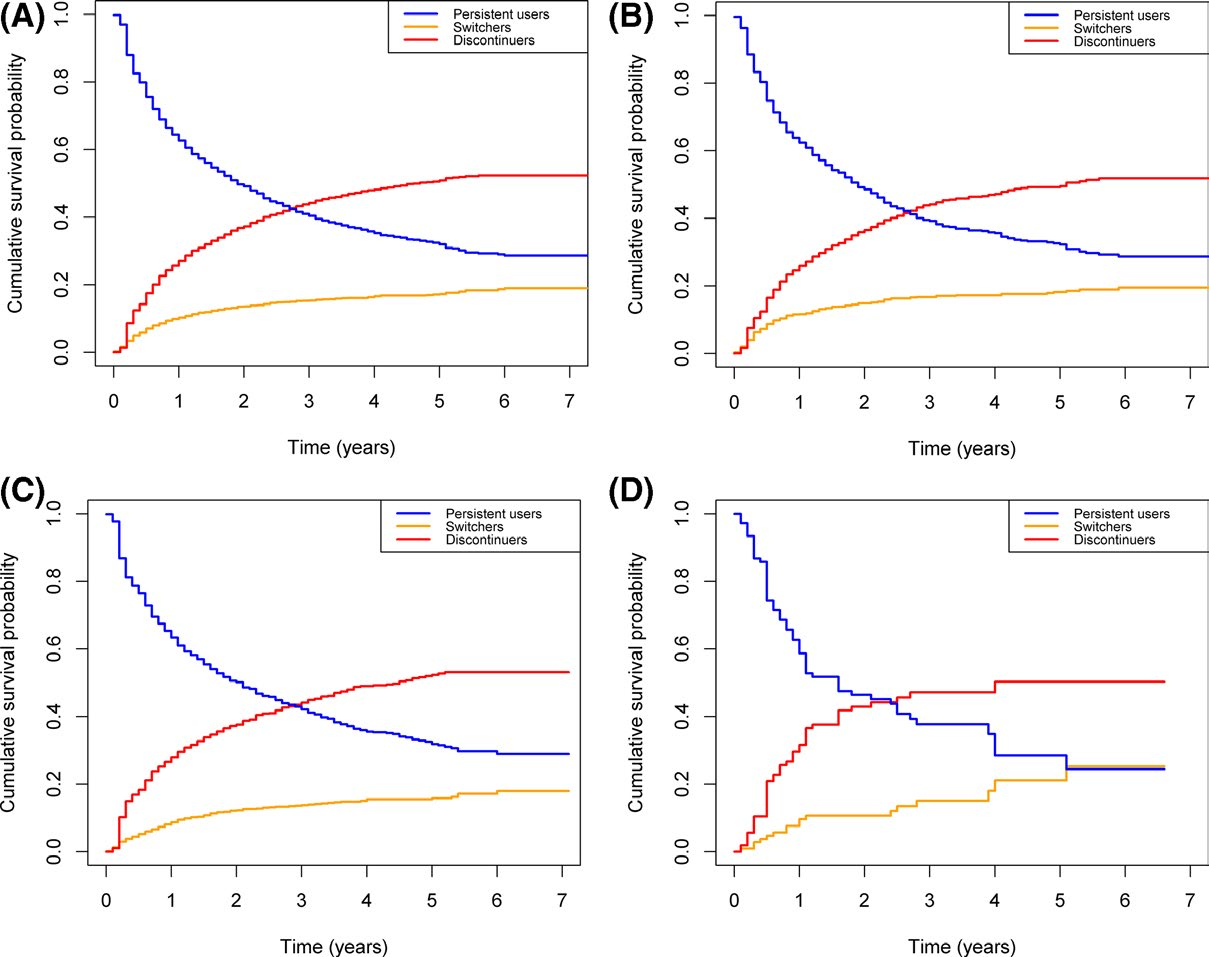
Kaplan–Meier curve of time of persistent use of initial TNFα inhibitor; time until switch to another biological; time until discontinuation TNFα inhibitor without switching for all indications (A), RD (B), IBD (C) and psoriasis (D)

Approximately 16% of patients switched from the initial TNFα inhibitor to another biological drug, which was comparable across indications, as shown in Figure 1. About 44.5% of patients discontinued their initial TNFα inhibitor without switching to another biological drug, this was comparable between the indications as well. One year after the index date, 62.4% of RD patients, 63.4% of IBD patients, and 58.7% of psoriasis patients were still using their index TNFα inhibitor. The median duration of use was 1.9 years in RD patients, 2.1 years in IBD patients, and 1.6 years in psoriasis patients.

**FIGURE 2:**
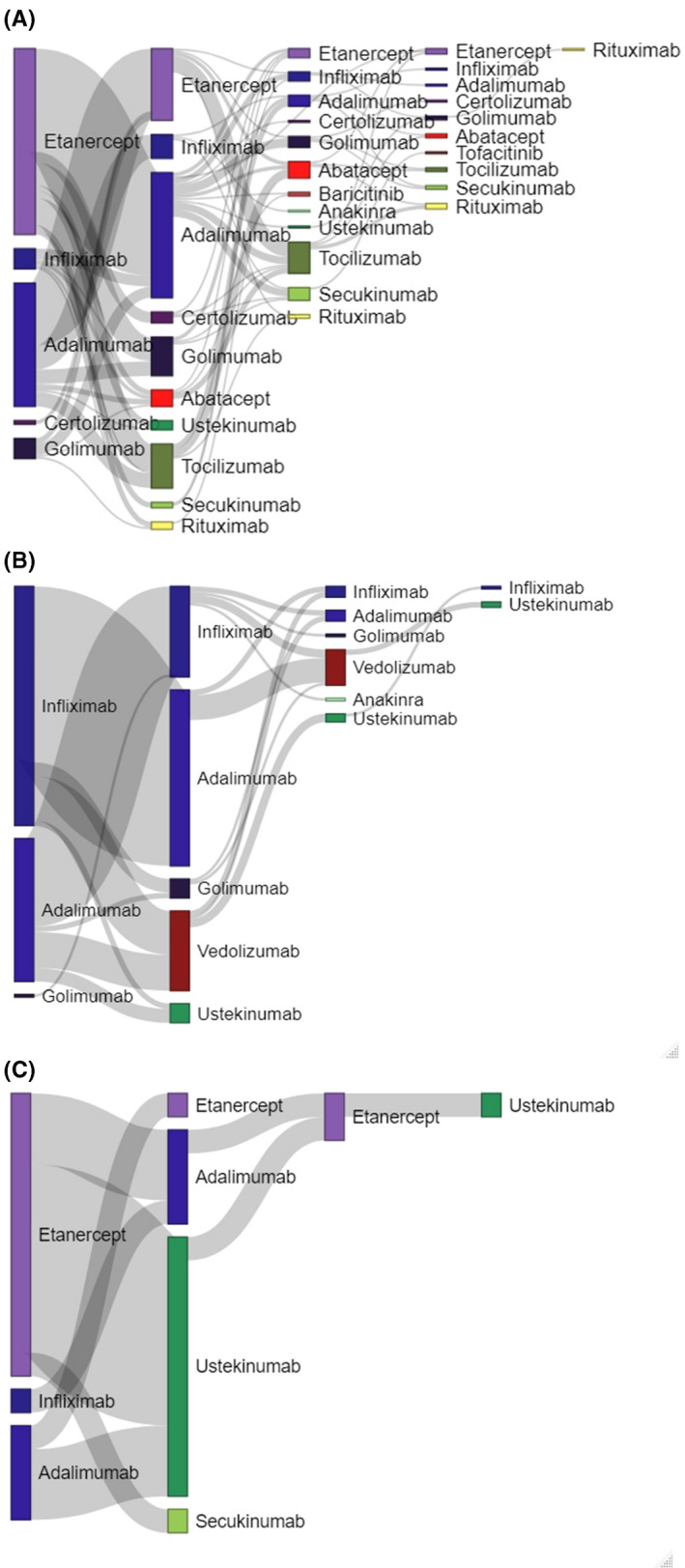
(A) Switching patterns of RD patients with median time (IQR) until switch. TNFα inhibitors (etanercept, infliximab, adalimumab, certolizumab, golimumab) were colored purple, selective immunosuppressants (abatacept, tofacitinib, baricitinib) were colored red, interleukin inhibitors (anakinra, ustekinumab, tocilizumab, secukinumab) were colored green and rituximab was colored yellow. (B) Switching patterns of IBD patients with median time until switch. TNFα inhibitors (infliximab, adalimumab, golimumab) were colored purple, selective immunosuppressants (vedolizumab) were colored red and interleukin inhibitors (anakinra, ustekinumab) were colored green. (C) Switching patterns of psoriasis patients with median time until switch. TNFα inhibitors (etanercept, infliximab, adalimumab) were colored purple and interleukin inhibitors (ustekinumab, secukinumab) were colored green

The majority of RD and IBD patients switched from their index TNFα inhibitor to a second TNFα inhibitor (76.6% and 74.3%); most psoriasis patients switched to ustekinumab (64.7%), as shown in Figure 2A–C. About 33% of RD patients, 20% of IBD patients, and 12% of psoriasis patients switched a second time; some patients to a third TNFα inhibitor (36.5% for RD and 37.0% for IBD), some to an interleukin inhibitor (41.3% for RD and 8.1% for IBD) and some to a selective immunosuppressant (19.0% for RD and 51.9% for IBD), except for psoriasis, these patients switched all to a TNFα inhibitor. Switching three times or more occurred in 8.9% of RD, 2.1% of IBD, and 5.9% of psoriasis patients. The median time until switch was comparable between patients with RD, IBD, and psoriasis (Figure 2A–C).

### 3.2 Determinants for switching

The assessment of determinants showed that patients suffering from RD who had a dose escalation of their TNFα inhibitor (OR 13.78, 95% CI 1.40–135.0) or initiated high‐dose corticosteroid treatment (OR 3.62, 95% CI 1.10–12.15) were more likely to switch biological treatment (Table 2).

**TABLE 2:** Determinants for the first switch to a second biological for RD patients

	No. cases *N* = 171	No. controls *N* = 627	OR (univariate) 95% CI	OR (multivariate) 95% CI

Median (IQR)age at index date	47.4 (29.5)	48.2 (26.3)	0.99 (0.98–1.00)	—
Gender				
Males	61 (35.7%)	241 (38.4%)	Ref	
Females	110 (64.3%)	386 (61.6%)	0.88 (0.81–1.60)	—
TNFα dose escalation				
No	168 (98.2%)	626 (99.8%)	Ref	
Yes	3 (1.8%)	1 (0.2%)	12 (1.25–115.4)*	13.78 (1.40–135.0)
Initiation/dose escalation immunomodulator				
No	143 (83.6%)	547 (87.2%)	Ref	
Yes	28 (16.4%)	80 (12.8%)	1.43 (0.85–2.42)	—
High‐dose corticosteroid				
No	166 (97.1%)	621 (99.0%)	Ref	
Yes	5 (2.9%)	6 (1.0%)	3.24 (0.99–10.65)*	3.62 (1.10–12.15)
Serum concentration measurement				
No	170 (99.4%)	627 (100%)		
Yes	1 (0.6%)	0 (0%)	NA	—

* *p*‐value <.1.

IBD patients who had a dose escalation of their TNFα inhibitor (OR 8.22, 95% CI 3.76–17.93), initiated or intensified immunomodulator treatment (OR 2.13, 95% CI 1.04–4.34), initiated high‐dose corticosteroid treatment (OR 6.91, 95% CI 2.81–17.01) or had a serum concentration measurement (OR 5.44, 95% CI 2.74–10.79) were more likely to switch as well (Table 3).

**TABLE 3:** Determinants for the first switch to a second biological for IBD patients

	No. cases *N* = 136	No. controls *N* = 459	OR (univariate) 95% CI	OR (multivariate) 95% CI

Median (IQR) age at index date	38.6 (31.8)	32.7 (31.9)	1.01 (0.99–1.02)	—
Gender				
Males	61 (44.9%)	204 (44.4%)	Ref	
Females	75 (55.1%)	255 (55.6%)	0.97 (0.66–1.43)	—
TNFα dose escalation				
No	91 (66.9%)	424 (92.4%)	Ref	
Yes	45 (33.1%)	35 (7.6%)	10.83 (5.51–21.26)*	8.22 (3.76–17.93)
Initiation/dose escalation immunomodulator				
No	95 (69.9%)	415 (90.4%)	Ref	
Yes	41 (30.1%)	44 (9.6%)	4.45 (2.65–7.89)*	2.13 (1.04–4.34)
High‐dose corticosteroid				
No	109 (80.2%)	440 (95.9%)	Ref	
Yes	27 (19.8%)	19 (4.1%)	8.12 (3.74–17.62)*	6.91 (2.81–17.01)
Serum concentration measurement				
No	86 (63.2%)	405 (88.2%)	Ref	
Yes	50 (36.8%)	54 (11.8%)	6.55 (3.65–11.77)	5.44 (2.74–10.79)

* *p*‐value <.1.

The study did not include a sufficient number of cases with psoriasis to allow for a case–control analysis in this group of patients.

The sensitivity analysis produced similar results both for the treatment patterns and determinants analysis as the main analysis (Table S1–S3).

## 4 DISCUSSION

In this study, we investigated switching patterns and determinants for switching in patients with RD, IBD, or psoriasis initiating treatment with TNFα inhibitors in the Netherlands between July 2012 and December 2017. Our study demonstrated that about 16% of patients switched biological treatment, mainly to another type of TNFα inhibitor. A limited number of patients (5.5% of the RD patients, 2.3% of the IBD patients and 1.9% of the psoriasis patients) switched twice during follow‐up. TNFα inhibitor dose escalation and initiation of high‐dose corticosteroid were associated with switching in RD patients while dose escalation of the TNFα inhibitor or immunomodulator, initiation of high‐dose corticosteroid treatment, and TNFα inhibitor serum concentration measurement were associated with switching in IBD patients.

Our study demonstrated that 16.6% of RD patients, 14.5% of IBD patients, and 16.0% of psoriasis patients switched biological treatment after a median of 0.52–1.96 years of use. Other studies with similar duration of follow‐up published similar percentages of switchers, ranging from 12.9% in RD and psoriasis patients to 14.6% in IBD patients.^14^, ^24^ A study in psoriasis patients reported higher percentage of switching (54.9%), which in part could be explained by the longer follow‐up of 12 years and inclusion of a biological drug that was withdrawn from the market.^25^ The majority of RD and IBD patients in our study switched to another type of TNFα inhibitor, which was in line with previous studies in these indications.^15^, ^26^

About 33% of RD patients, 19% of IBD patients, and 12% of psoriasis patients who switched once, additionally switched a second time during follow‐up. A similar switching rate to third‐line biological treatment of 20% in RD patients was found.^27^ In RD and IBD, no clear preference regarding the type of biological used for the second switch during follow‐up was seen. Surprisingly, 25 patients in our study sequentially used three different types of TNFα inhibitors, which is not in accordance with guidelines of the American College of Rheumatology and the European Crohn´s and Colitis Organisation.^28^, ^29^ However, until recently, particularly in IBD, limited options were available after the failure of treatment with TNFα inhibitors.

Our study showed that TNFα inhibitor dose escalation and initiation of high‐dose corticosteroid treatment was associated with an increased likelihood of switching to a second biological in both RD and IBD patients. Initiation or dose escalation of an immunomodulator and TNFα inhibitor serum concentration measurement were associated with switching as well in IBD patients. These factors are possible markers for disease worsening and, consequently, switching. In RD and IBD patients, disease flares are often treated by initiating high‐dose corticosteroids or immunomodulators.^28^, ^30^ However, in contrast to RD, if an IBD patient experiences a flare, measuring serum drug concentrations (and anti‐drug antibodies), and intensifying the dose are also commonly used strategies.^29^ Thus, in both indications, these determinants, together with the finding that switching occurred after a median of more than 6 months, might indicate that loss of effect of the index TNFα inhibitor, experienced as flaring of the disease was the most important reason for switching biological treatment. There is also some coherence between these actions as for a patient experienced a flare, a clinician could, for example, measure the TNFα inhibitor serum concentration and simultaneously initiate high‐dose corticosteroids to instantly treat the flare.

A study in IBD patients demonstrated that initiation of high‐dose corticosteroids and serum concentration measurement were predictors of switching.^15^ However, contradictory to our findings, dose escalation of the TNFα inhibitor was found to decrease the likelihood of switching. This discrepancy could be attributed to the authors’ more stringent definition of dose escalation compared to our study. We assessed dose escalation within a 60‐day time frame prior to switch while Chen et al. defined a dose escalation as a dose that was higher than the standard dose without using a specific timeframe. For example, if a patients was using etanercept once per 2 weeks, but increased the dose to once per 10 days in the 60‐day period prior to switching, we considered this a dose escalation.

Our study was, to the best of our knowledge, unique in mapping longitudinal switching patterns, including multiple switches, across the three major indications for TNFα inhibitor treatment and explored determinants for switching across multiple indications. Another strength of this study was the large number of included patients, which reflects the general patient population. Moreover, as patients were included from two large hospitals and one university hospital, this study provides an ideal reflection of switching patterns across various hospitals.

One of the three included hospitals had stringent guidelines for the first‐ and second‐line biological treatment for each indication; which possibly affected switching patterns. However, switching patterns for patients treated for RD and IBD at this hospital were similar to the other two hospitals who did not have stringent guidelines or restrictions. The local policies in one included hospital advised psoriasis patients not to initiate treatment with a TNFα inhibitor but with an interleukin inhibitor. Thus, we were only able to include a limited number of psoriasis patients from this hospital.

It is important to consider that patients might use the outpatient‐administered TNFα inhibitor differently from what is indicated on the dosing label. This could result in an overestimation of the number of discontinued patients. However, we applied a broad permissible gap of 90 days between dispensings to overcome this. Same applies to misclassification of first use, which we defined minimum biological‐free period of 6 months before the initiation. However, prolonging this period to 12 months did not impact our results.

We additionally did not have information on the reason for switching to another biological drug or the discontinuation of biological treatment. As the reason for switching treatment influences the choice of second‐line biological drug, this information might add to the understanding of the switching patterns seen.

Finally, as the indication for TNFα inhibitor treatment was derived from the specialism of the prescriber, we were unable to make distinctions between the individual RD, such as RA, AS, psoriatic arthritis, and juvenile idiopathic arthritis. RA and AS are the most prevalent rheumatic diseases,^31^ we believe that these are also the most prevalent types of RD in our cohort. As the biological treatment strategies in RA and AS are comparable and there are no differences in reimbursement regulations between these indications, we believe that aggregating all types of RD has little impact on our results.

In conclusion, this large study of real‐life data on biological use demonstrated‐specific switching patterns of patients who initiated TNFα inhibitor treatment. Approximately 16% of patients switched biological treatment, this was comparable between the three indications. Most RD and IBD patients switched to another TNFα inhibitor. A minority of the patients switched a second time, but in these patients, there was no clear preference for TNFα inhibitors or biological drugs belonging to another mechanistic class.

TNFα inhibitor dose escalation and the initiation of high‐dose corticosteroid treatment were determinants for switching in RD patients. TNFα inhibitor dose escalation, immunomodulator dose escalation, the initiation of high‐dose corticosteroid treatment and the measurement of TNFα inhibitor serum concentration were determinants for switching in IBD patients. These findings might help clinicians to anticipate on switching of TNFα inhibitor treatment in these patients.

## DISCLOSURE

The authors declare they have no conflict of interest.

## ETHICAL APPROVAL

The Institutional Review Board of the Spaarne Gasthuis approved the study protocol. This study was additionally reviewed by the Medical Research and Ethics Committee UMC Utrecht (protocol reference number 19‐049/C), which concluded that ethical approval was not required, as the study did not fall under the scope of the Dutch Medical Research Involving Human Subjects Act.

## Supporting information

Table S1‐S3

## Data Availability

Research data will not be shared.
