# Supplementary material for: Switching TNFα inhibitors: Patterns and determinants
Source: Pharmacol Res Perspect. 2021 Jul 24;9(4):e00843. doi: 10.1002/prp2.843 (PMC8305431; doi:10.1002/prp2.843)
Supplement: Supplementary file 1 — Table S1‐S3 [file PRP2-9-e00843-s001.docx]

## Supplementary files

Table S1: Number of switched and discontinued patients – sensitivity analysis

|  | Total  (n=2010) | RD (n=1027) | IBD  (n=883) | Psoriasis (n=100) |
| --- | --- | --- | --- | --- |
| No. switched patients (%) | 306 (15.2%) | 161 (15.7%) | 129 (14.6%) | 16 (16%) |
| No. discontinued patients (%) | 879 (43.7%) | 440 (42.8%) | 393 (44.5%) | 46 (46%) |

Table S2: Patients on index TNFα inhibitor after one year and median duration of use – sensitivity analysis

|  | Total  (n=2010) | RD (n=1027) | IBD  (n=883) | Psoriasis (n=100) |
| --- | --- | --- | --- | --- |
| No. Patients on index TNFα inhibitor after one year (%) | 1255 (62.4%) | 626 (61.8%) | 571 (63.6%) | 56 (57.2%) |
| Median duration of use (year) | 1.9 | 1.9 | 2.0 | 1.6 |

Table S3a: Determinants for the first switch to a second biological for RD patients – Sensitivity analysis

|  | No. cases  N= 153 | No. controls  N= 533 | OR (univariate)  95% CI | OR (multivariate)  95% CI |
| --- | --- | --- | --- | --- |
| Median (IQR)  Age at index date | 46.5 (29.1) | 48.8 (28) | 0.99 (0.98-1.01) | - |
| Gender  Males  Females | 56 (36.7%)  97 (63.3%) | 225 (42.2%)  308 (57.8%) | Ref  0.79 (0.88-1.84) | - |
| TNFα dose escalation  No  Yes | 149 (97.4%)  4 (2.6%) | 532 (99.8%)  1 (0.2%) | Ref  13 (1.43-121.7)* | - |
| Initiation/ dose escalation immunomodulator  No  Yes | 131 (85.6%)  22 (14.4%) | 480 (90.1%)  53 (9.9%) | Ref  1.56 (0.90-2.72) | - |
| High-dose corticosteroid  No  Yes | 151 (98.7%)  2 (1.3%) | 529 (99.2%)  4 (0.8%) | Ref  1.9 (0.35-10.4) |  |
| Serum concentration measurement  No  Yes | 152 (99.3%)  1 (0.7%) | 532 (99.8%)  1 (0.2%) | Ref  4 (0.25-63.96) | - |

*p-value <0.1

Table S3b: Determinants for the first switch to a second biological for IBD patients – Sensitivity analysis

|  | No. cases  N= 125 | No. controls  N= 426 | OR (univariate)  95% CI | OR (multivariate)  95% CI |
| --- | --- | --- | --- | --- |
| Median (IQR)  Age at index date | 38.8 (31.4) | 33.8 (32.9) | 1.00 (0.99-1.02) | - |
| Gender  Males  Females | 60  65 | 198  228 | Ref  0.95 (0.64-1.42) | - |
| TNFα dose escalation  No  Yes | 99  26 | 402  24 | Ref  11.1 (4.45-27.55)* | 13.66 (4.73-39.43) |
| Initiation/ dose escalation immunomodulator  No  Yes | 86  39 | 382  44 (%) | Ref  4.70 (2.69-8.23)* | 4.04 (2.02-8.06) |
| High-dose corticosteroid  No  Yes | 27  98 | 406  20 | Ref  9.59 (4.08-22.52)* | 10.68 (4.73-39.43) |
| Serum concentration measurement  No  Yes | 76  49 | 373  53 | Ref  6.65 (3.66-12.09)* | 5.03 (2.56-9.12) |

*p-value <0.1
